# The Story of the Insane from Year to Year

**Published:** 1889-08-03

**Authors:** 


### The Story of the Insane from Year to Year.

Asylum Reports.

### NORTH RIDING ASYLUM.

Yorkshire is well off for asylums, having not fewer than six institutions for the insane, supported by the rates, and the Friends' Retreat and Bootham Hospital are more or less of a charitable nature. The North Riding Asylum is situated near York. On the last day of 1888 it had 622 patients in residence, being an increase of 13 on the numbers for 1887. The recovery-rate was 39·7, and the death-rate 9·1 per cent., both calculated in the way usual in asylums. The admissions amounted to 141. Not fewer than 13 of these were general paralytics, and 23 were hopelessly demented. In 36 the disease was known to be hereditary, in 11 it was said to be owing to religious excitement, and in 9 drink was the assigned cause. Of the deaths 8 were due to phthisis, and 15 to general paralysis, two women being among the latter. Many additions and alterations are in progress, or have been carried out during the year, the most important being a new block for 50 women, and a messroom for the nurses. We notice that the matron retired on a well-earned pension of £80, after seventeen years' service, and one of the nurses followed suit with a pension of £30. The weekly cost of maintenance is 9s. 2d. A ground plan of the asylum is given in this year's report, and very useful it is for reference and comparison.

### OMAGH DISTRICT ASYLUM.

To this institution patients from the counties of Tyrone and Fermanagh are admitted. It contained 519 cases on 31st December, 1888. During the year there were 165 admissions, 131 discharges, and 42 deaths. These numbers show that the percentage of recoveries was 44·2 and the deaths 7·8. The former is high and the latter low. Dr. Carre says, "We had only two attempted escapes during the year, which shows that the attendants were careful of the patients committed to their charge." Perhaps, but it may show that the freedom of the patients is curtailed. The male side of the asylum is full, and recourse was had to the old expedient of discharging patients to the workhouse — an expedient of which we cannot approve, and which most likely will only give temporary relief. The enlargement of the asylum is, however, under consideration. There is no report by the Visiting Committee; but there are two by the lunacy inspectors who would seem to visit half-yearly in Ireland. One of these reports runs to two lines, and the other to thirteen lines. Both are favourable to the condition of the asylum.

### WARWICK COUNTY ASYLUM.

The admissions number 171, being higher than in any previous year. Many of the cases were most unpromising. We notice that 25 were epileptics, 10 were idiots, 8 were paralytics, and 20 were suffering from chronic forms of insanity. The year closed with 684 patients in the asylum; 81 patients were discharged, and 60 died. The recoveries were 33·5 per cent., and the deaths were 9·1 per cent. Four of the deaths were from dysentery, one from enteric fever, and one from intestinal ulceration. Dr. Sankey says, "Seven cases of dysentery and typhoid fever have occurred on the male side. I am at a loss to account for the outbreak, unless it be that there is insufficient room for the number of patients resident, and insufficient means of at once isolating cases of diarrhœa immediately on its appearance." On the resignation of the matron a great improvement was carried out in the staff by substituting a head nurse and a housekeeper. The former receives £60 a year and the latter £50. The carpenter retired on a pension of £44 a year. We congratulate him and hope he will have many years of life remaining in which to enjoy his well-earned rest.

### WATERFORD DISTRICT ASYLUM.

On the last day of 1888 there were 332 patients resident, which would seem to be 32 in excess of what the asylum is intended to accommodate. Sixty-eight patients were admitted during the year, 36 were discharged, and 22 died; 48·52 per cent. were discharged recovered, and 6·19 per cent. died, both calculated in the usual manner. In 12 cases the insanity was caused by hereditary transmission, and in 8 it was owing to drink. We did not notice any table showing the causes of death. One of the inspectors states: "The house in detail is in good working order, and the provisions which I examined of excellent quality. The farm, too, appear to be well managed, and with an abundant produce."

### BERKS COUNTY ASYLUM AT MOULSFORD.

This asylum was opened in 1870, and at the end of that year it contained only 111 patients, whereas now there are 496, an instance among many others of how the registered insane have grown during the last decade or two. During 1888 the admissions reached 102, the discharges were 47, and the deaths 41. The recovery rate stands at 37·2 per cent., and the death-rate at 8·3 per cent. The weekly cost of maintenance was 8s. 6d. On referring to the pensions question, the visitors have the following remarks, which should be engraved in every committee room: "During the past summer, whilst the present Local Government Act was being discussed in the House of Commons, the committee of visitors passed a resolution urging upon Parliament the justice of inserting in that Act the claims of existing officers of lunatic asylums to receive pensions, either in the case of failure of health, or should they be over 60 years of age and have served for 15 years, and the committee consider that in such cases the right to receive such a pension as is allowed by the existing Lunacy Acts should be assured to those who have spent the best years of their lives in the public service, and in a harassing and anxious occupation." Assuredly, however, the age stated should be 50 instead of 60. What would be the use of an attendant or nurse at the age of 60?

### NORTHAMPTON COUNTY ASYLUM.

On the last day of 1888 the numbers were 643, showing a decrease of 61, this being caused by the transference of a detachment of Essex patients received under contract, and removed on the opening of the new block at the Brentwood Asylum. There were 213 admissions, 187 discharges, and 87 deaths. Of the discharges, 26·3 were recovered, but, excluding transfers, this percentage would be raised to 30. The medical superintendent states in his report that "the tendency at the present day is almost everywhere to send more harmless imbeciles to asylums than used to be the case, and the medical superintendent believes that this is the right course for the union authorities to pursue where the friends of the patient are not both able and willing to look properly after him. The influx of these cases will reduce the percentage of recoveries; but, nevertheless, there can hardly be a doubt that more curable cases are cured now than in any former time." The death-rate is a little over 12 per cent. on the average number resident. This asylum is the only one of its kind having a separate block for the treatment of idiot children. It was opened during the year, and contained 19 cases. We agree with Mr. Greene, when he says that "one of the greatest wants of the present day is a private asylum receiving patients at rates between 10s. and 20s. a week." There is no reason why this should not be done, and provided the asylum contained a sufficient number of beds we have no doubt it would answer in every way.

